# Symbiosis maintenance in the facultative coral, *Oculina arbuscula*, relies on nitrogen cycling, cell cycle modulation, and immunity

**DOI:** 10.1038/s41598-021-00697-6

**Published:** 2021-10-27

**Authors:** H. E. Rivera, S. W. Davies

**Affiliations:** Department of Biology, Boston University, Boston, MA USA

**Keywords:** Marine biology, Molecular ecology

## Abstract

Symbiosis with unicellular algae in the family Symbiodiniaceae is common across tropical marine invertebrates. Reef-building corals offer a clear example of cellular dysfunction leading to a dysbiosis that disrupts entire ecosystems in a process termed coral bleaching. Due to their obligate symbiotic relationship, understanding the molecular underpinnings that sustain this symbiosis in tropical reef-building corals is challenging, as any aposymbiotic state is inherently coupled with severe physiological stress. Here, we leverage the subtropical, facultatively symbiotic and calcifying coral *Oculina arbuscula* to investigate gene expression differences between aposymbiotic and symbiotic branches within the same colonies under baseline conditions. We further compare gene ontology (GO) and KOG enrichment in gene expression patterns from *O. arbuscula* with prior work in the sea anemone *Exaiptasia pallida* (Aiptasia) and the salamander *Ambystoma maculatum*—both of which exhibit endophotosymbiosis with unicellular algae. We identify nitrogen cycling, cell cycle control, and immune responses as key pathways involved in the maintenance of symbiosis under baseline conditions. Understanding the mechanisms that sustain a healthy symbiosis between corals and Symbiodiniaceae algae is of urgent importance given the vulnerability of these partnerships to changing environmental conditions and their role in the continued functioning of critical and highly diverse marine ecosystems.

## Introduction

Symbiotic relationships are ubiquitous across the tree of life. Interactions between species can allow an organism to access resources such as nutrients, amino acids, vitamins, or energetic reserves that may be otherwise unattainable^1–3^. Bacteria within the guts of mammals and insects can influence health, growth, and even reproduction^4–6^. Fungi living in the roots of legumes allow them to access key nutrients like nitrogen or phosphorus^7^. *Vibrio* in specialized squid organs enable bioluminescence that facilitates predator avoidance^8^. Studying symbiotic relationships can elucidate important metabolic, immunological, and evolutionary mechanisms that allow species to develop novel ecological niches^2,3,9^. The obligate nature of many symbioses, however, can make it difficult to disentangle the mechanisms that maintain these relationships from those that underpin a stress-induced breakdown of the symbiotic relationship itself.

This breakdown of symbiosis has been especially devastating for one iconic example of such symbioses: the relationship between reef-building corals and their unicellular dinoflagellate partners from the family Symbiodiniaceae^10^. These photosynthetic endosymbionts live within coral tissues and can provide upwards of 90% of their coral host’s energetic needs^11^. As such, most coral species cannot survive without their symbionts for long periods of time^12^. Unfortunately, coral-dinoflagellate symbioses quickly break down under elevated temperatures in a process known as coral bleaching, which has been devastating coral reef ecosystems around the globe as ocean temperatures continue to rise^13,14^.

There has been extensive study of the physiological and molecular (e.g. gene expression) responses of coral-algal symbioses under heat, low pH, and other environmental stressors (e.g.^15–18^). These studies have consistently implicated several key pathways in the coral-algal stress response. For instance, oxidative stress response genes such as superoxide dismutase are consistently upregulated under stress^19–21^, as are apoptotic response genes like tumor necrosis factors, ubiquitin, and heat response genes such as heat shock proteins^22–25^. While these studies have been invaluable in building our understanding of an ecologically important environmental stress response, our knowledge of the mechanisms underpinning the establishment and maintenance of symbiosis under homeostatic conditions has lagged, since for most coral species an aposymbiotic state is inherently coupled with stress.

**Figure 1:**
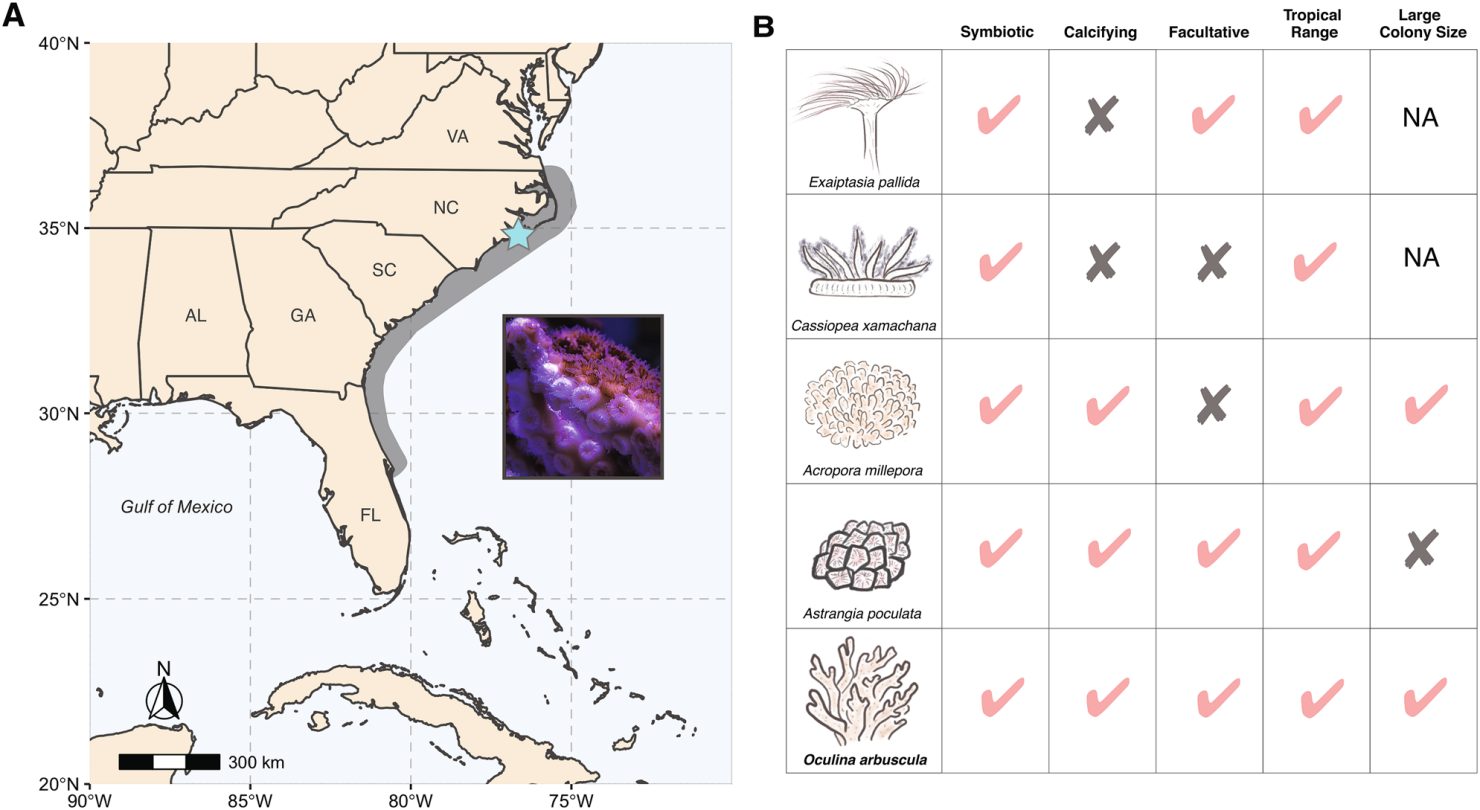
(**A**) Map of *Oculina arbuscula* range distribution (grey), with sampling site denoted by a blue star. Inset: photograph highlighting both symbiotic (brown, top right) and aposymbiotic (white, bottom left) polyps of the same *O. arbuscula* colony (Photo credit: Brooke E. Benson). Map was created using the rnaturalearth v.0.1.0 R package (https://cran.r-project.org/package=rnaturalearth) (**B**) Comparison of key attributes for the study of dysbiosis among various cnidarian model species. Species drawings by H.E. Rivera (first author).

To circumvent these limitations, the majority of prior studies have turned to examining the early life stages of coral larvae that are born without symbionts (e.g.^26–28^) or other symbiosis models such as the sea anemones *Exaiptasia pallida* (Aiptasia) or *Anthopleura elegantissima*, which can be maintained in an aposymbiotic states under laboratory conditions (e.g.^29–32^). Work in these systems has uncovered key mechanisms for control of symbiont densities^33–35^, lectin and other proteins involved in symbiont recognition and endocytosis^36,37^, as well as differences in the repression of the host immune system by symbionts of different genera^38^. Still, much can be learned from studying adult calcifying corals in a naturally aposymbiotic state. There are 12 species of calcifying corals that are considered facultatively symbiotic with Symbiodiniaceae, meaning they can survive without symbionts in a non-stressed state^39^. These species tend to have sub-tropical distributions and can be found with extremely low symbiotic densities in the wild, especially at more northern latitudes, in low light environments, or in winter seasons^40,41^. Unlike other symbiosis models, such as *Aiptasia* or the upside-down jellyfish *Cassiopea xamachana*, facultative corals including *Oculina arbuscula* (this study) and *Astrangia poculata* (another emerging model species^42–44^) calcify just as tropical reef-building corals do (Fig. 1B). Given the high metabolic costs associated with calcification, facultative species may serve as more representative models for the impacts of dysbiosis on the ability of corals to grow and maintain net ecosystem calcification in the face of warmer and more acidic oceans^45^.

Here, we examined gene expression differences between naturally symbiotic and aposymbiotic branches of the facultative coral, *O. arbuscula.* This coral, which inhabits reefs from North Carolina to central Florida, hosts *Breviolum psygmophilum* as its almost exclusive algal symbiont^46^ (Fig. 1). Colonies can be found with both symbiotic (high symbiont density, brown color) and aposymbiotic (low symbiont density, white color) branches or as entirely symbiotic and aposymbiotic colonies in the wild. Here, we generate the first transcriptome for *O. arbuscula* and investigate gene expression differences between branches of differing symbiotic states with the goal of identifying mechanisms that are responsible for symbiosis maintenance as opposed to stress-induced symbiosis breakdown. We further compare our results across other taxa including Aiptasia and a symbiotic vertebrate, the salamander *Ambystoma maculatum*, which hosts endosymbiotic *Chlorella* cells during its embryonic stages.

## Methods

### Coral colony collection and sampling

Six colonies of the facultatively symbiotic coral *Oculina arbuscula* Agassiz, 1864 were collected from 15–20 ft depth at Radio Island Jetty, North Carolina (34° 42.520′ N, 76° 40.796′ W; Fig. 1) using a hammer and chisel on May 25, 2018. All *O. arbuscula* colonies were collected under the NC Division of Marine Fisheries Permits #706481 and #1627488. Corals were transported overnight to the Davies Marine Population Genomics Lab at Boston University and maintained in aquaria at 25 °C (± 0.17, SD), 34.6 PSU (± 0.76, SD), and pH of 8.0 (± 0.08, SD) for two months. Because symbiont density varied substantially among branches of the same colonies, two tissue biopsies were collected from each colony (labeled A–F)—one from a branch with high symbiont density (brown in color, termed symbiotic) and another from a low symbiont density branch (pale or white in color, termed aposymbiotic). Colonies were preserved in 200-proof ethanol and maintained at – 80 °C until RNA isolation. This sampling yielded a total of twelve unique transcriptomic libraries (colonies A–F, with symbiotic and aposymbiotic branches).

### RNA extraction and sequencing

RNA was extracted using the Invitrogen™ RNAqueous™ Total RNA Isolation Kit, with the addition of a DNase step (Invitrogen rDNase I) to remove genomic DNA contamination. Extracted total RNA was quantified on Denovix spectrophotometer and ran on a 1% agarose gel to visualize RNA integrity. RNA quantities were normalized and sent to NovoGene where libraries were prepared for RNAseq using poly-A capture and cDNA synthesis and amplification. Resulting libraries were sequenced on an Illumina HiSeq4000 with 150 bp paired-end reads. Sequencing depth ranged from 9.8 to 13.3 million reads per sample, with average quality ranging from 40 to 42.

### Holobiont transcriptome assembly (script: transcriptome_assembly.sh)

A total of 277 million paired-end reads were quality filtered and trimmed using Trimmomatic through Trinity (v.2.6.6)^47^ with default Trimmomatic parameters and using the Illumina TruSeq paired end adapters (“TruSeq-PE-2”) file. Reads < 25 bases long after filtering were removed. These steps filtered 6158 (< 1%) of PE reads, 7412 (< 1%) of forward reads, and 9,086,121 (3.28%) of reverse reads. A total of 268,009,326 (96.72%) reads were used as input for de novo holobiont transcriptome assembly using Trinity (v.2.6.6) with default parameters and in-silico normalization of reads. Contigs shorter than 500 base pairs in length after assembly were removed (58%: 783,085 of 1,346,349 contigs). The quality metrics of the resulting holobiont transcriptome assembly fasta file is reported in Table 1.

**Table 1:** Transcriptome assembly statistics for *Oculina arbuscula* and *Breviolum psygmophilum* transcriptomes, as well as unparsed “holobiont” transcriptome for reference.

Transcriptome	# Contigs	N50 (bp)	L50	Max length (bp)	Average length (bp)	BUSCO%	%GC
*Oculina arbuscula*	57,470	1678	14,552	32,901	1418	72.1	42.8
*Breviolum psygmophilum*	31,970	1458	9764	8436	1290	44.4	51.4
Holobiont	563,264	1208	152,890	32,901	1092	95.6	41.8

BUSCO% scores represent the sum of percentages of single and duplicate copy orthologs against the Eukaryota core gene set. Note that the “holobiont” statistics are not simply the sum of the coral and symbiont transcriptomes as this “holobiont” was filtered to generate unambiguous coral and symbiont fractions (see “Methods”).

### Separation of coral host and algal symbiont transcripts (script: transcriptome_host_sym_fraction.sh)

Four custom databases (cnidarian-holobiont, *in hospite* Symbiodiniaceae, aposymbiotic cnidarian data only, and cultured *ex hospite* Symbiodiniaceae data) previously described in^15^ were used to filter and designate contigs as host or symbiont origin. Contigs were aligned to these databases using BLAST (v.2.7.1), in tblastx mode (E-value of 0.001, maximum of five target sequences). BLAST results were parsed to pull matches that were at least 100 bp in length and minimum of 80% sequence identity. Coral-assigned transcripts (*N* = 99,549) consisted of contigs blasting to the coral holobiont database (*N* = 109,061) with any contigs that also mapped to the *ex hospite* symbiont database removed (*N* = 9512). Symbiont-assigned transcripts (*N* = 42,035) were generated from contigs mapping to the *in hospite* Symbiodiniaceae database (*N* = 47,611) with contigs mapping to the aposymbiotic cnidarian database removed (*N* = 5576). Any contigs assigned to both host and symbiont fractions after these steps were also subsequently removed from both assemblies (*N* = 6093 reads).

Microbial rRNA contamination in both symbiont and host transcriptome fractions was identified by blasting against the SILVA database (132 LSUParc and SSURef_Nr99 combined—downloaded Dec 2017; E-value of 0.05, gap open penalty of 5, and gap extend penalty of 2, and penalty of 3). Contigs assigned to SILVA sequences were removed (*N* = 78 from coral transcriptome; *N* = 48 from symbiont transcriptome).

### Collapsing of isoforms, transcript annotation, and transcriptome quality assessment (script: collapse_annotate_clean_transcriptomes.sh, busco.sh)

Highly similar isoforms generated from the Trinity assembly were further collapsed to keep only the longest isoform as a representative using CD-HIT^48^ (parameters: -c 0.99 -G 0 -aL 0.3 -aS 0.3) as differential abundance estimation has been shown to be more accurate at the gene versus isoform level^49^. The resulting coral (*N* = 58,311 contigs) and symbiont (*N* = 31,982 contigs) transcriptomes were then annotated through blastx against the uniprot database (downloaded June 12, 2019), with an E-value of 0.001. Genes that were annotated as “chroloplastic” or “chlorophyll” were removed from the coral transcriptome (*N* = 93). Genes annotated to *Acropora millepora* or *Nematostella vectensis* were removed from the symbiont transcriptome (*N* = 12). Transcriptome statistics were assessed using the BBTools (https://sourceforge.net/projects/bbmap/) ‘stats.sh’ script with the coral and symbiont transcriptome files as inputs. Gene names and gene ontology categories were extracted from the blast results files using custom scripts (see data accessibility statement). To quantify transcriptome completeness, the coral and symbiont transcriptomes were compared against the BUSCO eukaryota_ob10, metazoa_ob10 (coral only), and alveolata_ob10 (symbiont only) databases using BUSCO v.4.0.5.

### Identifying *O. arbuscula* clones (scripts: oculina_rna_snps.sh and oculina_rna_snps.R)

Branching corals are known to reproduce asexually through fragmentation^50^. To identify potential *O. arbuscula* clones, we called single nucleotide polymorphisms (SNPs) from RNA-sequencing reads. Reads from symbiotic and aposymbiotic branches of the same colony were concatenated and mapped to the assembled host transcriptome using bowtie2^51^, allowing up to five alignments per read (− k = 5).

Bam alignment files were merged using samtools^52^ with read groups used to identify colony origin (-rh option). Samtools/bcftools were used to generate a vcf file of reads aligned to the transcriptome (-mpileup) and to index the transcriptome and merged reads files (-faidx). GATK haplotyper (Poplin et al. 2017) was then used to identify SNPs using default parameters. The resulting vcf file was then filtered using vcftools^53^ to retain loci where all six colonies had at least 10 × coverage, with a minimum quality score of 30. Lastly, indels were removed and only bi-allelic SNPs were retained, generating 29,633 SNPs.

The final vcf file was imported into R and the packages *adegenet*^54^ and *ape*^55^ were used to calculate a pairwise genetic distance matrix using the dist.gene() function in *ape* and hierarchical clustering using hclust(). Two colonies (colony A and E) were identified as clones with exceptionally long branch lengths separating them from all other colonies (Figure S7). To confirm dendrogram results, a Principal Component Analysis in *adegenet* also showed that colonies A and E strongly clustered together across several PC pairs (Figure S8). Colony E was therefore randomly chosen and removed from downstream differential gene expression analyses described below. It should be noted, however, that including both samples while accounting for genotype in the DESeq model or removing colony A instead of E did not influence final results and both samples had similar numbers of raw reads (45 vs. 43 million paired reads) and percentage of reads mapping to the coral host transcriptome (96% and 97% for aposymbiotic samples and 86% and 87% for aposymbiotic samples of colonies A and E, respectively; Table S1).

### Read mapping and gene expression analyses (scripts: read_mapping.sh, deg_analyses.R, deg_analyses.html)

Assembled *Oculina arbuscula* (coral) and *Breviloum psygmophilum* (symbiont) transcriptome fasta files and associated isogroup files were concatenated for read mapping. Trimmed paired-end reads from each sample were mapped to the concatenated holobiont transcriptome using bowtie2 to report the best of all possible alignments. A custom Perl script (samcount.pl) was used to count the number of reads mapping to coral or symbiont contigs. Counts files were then imported into R v. 3.6.1 for further analyses.

Expression data were inspected for outliers using the *arrayqualitymetrics* package^56^ and no outliers were detected. Only genes with at least 10 counts across all samples were retained (*N* = 25,428). Differentially expressed genes (DEGs) were identified using DESeq2 v. 1.26.0^57^ in R, with the model: *design* =  ~ *Genet ID* + *SymbioticState*. A contig was considered significantly differentially expressed if it had an FDR adjusted p-value < 0.1.

Data were normalized using the rlog transformation function in DESeq2. Normalized data were analyzed via principal components using the prcomp() function in R. The effect of genet and symbiotic state were tested with a PERMANOVA using the adonis() function in the R package *vegan*^58^ with Euclidean distances between samples. A heatmap of significantly differentially expressed genes was generated with the *pheatmap* R package^59^.

### Gene ontology enrichment analyses (scripts: deg_analyses.R and deg_analyses.html)

To determine whether global gene expression patterns showed enrichment of different gene ontology (GO) classes, the collection of scripts ‘GO_MWU’ from^60^ was used (https://github.com/z0on/GO_MWU). Here, the gene ontology database (go.obo v.1.2) was used to test for enrichment of GO terms based on the ranked − log signed p values of each gene. Gene ontology terms that were over-represented or under-represented were then visualized in a tree format that groups GO terms with other terms with similar functions.

### EuKaryotic Orthologous Classes (KOG) and GO enrichments comparisons among taxa (scripts: deg_analyses.R and deg_analyses.html)

To compare our results to other gene expression datasets investigating symbiotic and aposymbiotic states under baseline conditions, we conducted a KOG enrichment analysis on our data and two other datasets: the sea anemone *Exaiptasia pallida*^61^ and the salamander *Ambystoma maculatum*^62^. KOG classes represent high level conserved functions of orthologous genes across eukaryotic taxa, thereby facilitating the comparison of functional characteristics of transcriptomic responses across the tree of life ^63^. KOG annotations for all datasets were obtained through eggNOG-mapper v.2 (http://eggnog5.embl.de/#/app/home). KOG enrichment analyses were performed with the *KOG_MWU* package in R^64^. This analysis is analogous to GO_MWU but operates on KOG classes instead of GO terms.

In addition, we also conducted GO_MWU enrichment analyses for both *E. pallida* and *A. maculatum* datasets. There were no significantly enriched GO terms for *A. maculatum*, which is consistent with the authors original findings^62^, so only comparisons between *E. pallida* and *O. arbuscula* are included here. Both KOG and GO comparisons among datasets compare the delta ranks of the terms, which is the difference between mean rank (in the list of signed log p values) of genes belonging to a KOG/GO class and mean rank of all other genes. GO terms that were significant (p < 0.05) in both *E. pallida* and *O. arbuscula* datasets were compared for overlap.

## Results and discussion

### Generation of high quality *Oculina arbuscula* and *Breviolum psygmophilum* transcriptome assemblies

After adapter trimming and quality filtering, a total of 277,102,859 reads were retained (99.99%), of which 268,009,326 were paired (96.72%) and 9,086,121 were unpaired (3.8%). The assembled holobiont metatranscriptome had a total of 563,264 contigs (N50 = 1280). Of these, 57,470 were unambiguously assigned as *O. arbuscula*-specific contigs, with an average length of 1418 bp and an N50 of 1678 (Table 1). Among coral host contigs, 19,954 (34%) had gene annotations based on sequence homology. These results are similar to other coral transcriptomes with N50 in the low thousands and several tens of thousands of contigs^15,65^. Genes in the host transcriptome matched 72.1% of the core eukaryotic gene set in the BUSCO database (Table 1; Fig S1). We unambiguously assigned 31,970 contigs as *B. psygmophium* specific, with an average length of 1290 bp and an N50 of 1458 (Table 1). Among symbiont contigs, 11,932 (37%) had gene annotations. The symbiont transcriptome had a higher fraction of missing genes from the eukaryota (46%) BUSCO gene set than the coral transcriptome (5%), though matches to the alveolata (missing 31%) BUSCO gene set were better (Figure S1). While BUSCO recommends using the most closely related database to assess completeness, we include eukaryota results to contrast coral and symbiont transcriptomes. Genes are likely to differ in expression by symbiotic states, and this is expected for the algal symbiont given that it completely loses its flagellated state and lives within the coral cell in the symbiosome^66^.

All raw reads are archived in the National Center for Biotechnology Information Short Read Archive (SRA) under BioProject number PRJNA746598, transcriptome assembly and annotation files are available at http://sites.bu.edu/davieslab/data-code/, and scripts for read processing, transcriptome assembly, and holobiont parsing, are available at https://github.com/hrivera28/Oculina_arbuscula_transcriptome.

Average sample mapping efficiency to the concatenated host and symbiont transcriptomes was 48%, with higher mapped reads to *O. arbuscula* transcripts observed in aposymbiotic samples (average 93%) when compared to symbiotic samples (average 75%) (Figure S2). Assignment to *B. psygmophilum* transcripts was much lower in aposymbiotic relative to symbiotic samples, consistent with the assumption that symbiont densities were significantly lower in aposymbiotic branches (Table S1; Figure S2; *p* = 0.012*, N* = 6, one-tailed paired t test). Colony E (clonal with colony A) was included in this analysis as it represents an independent sample of aposymbiotic/symbiotic branches within a colony, hence *N* = 6 here instead of *N* = 5 for most other analyses.

### Gene expression was strongly modulated by genet with secondary influence of symbiotic state

**Figure 2:**
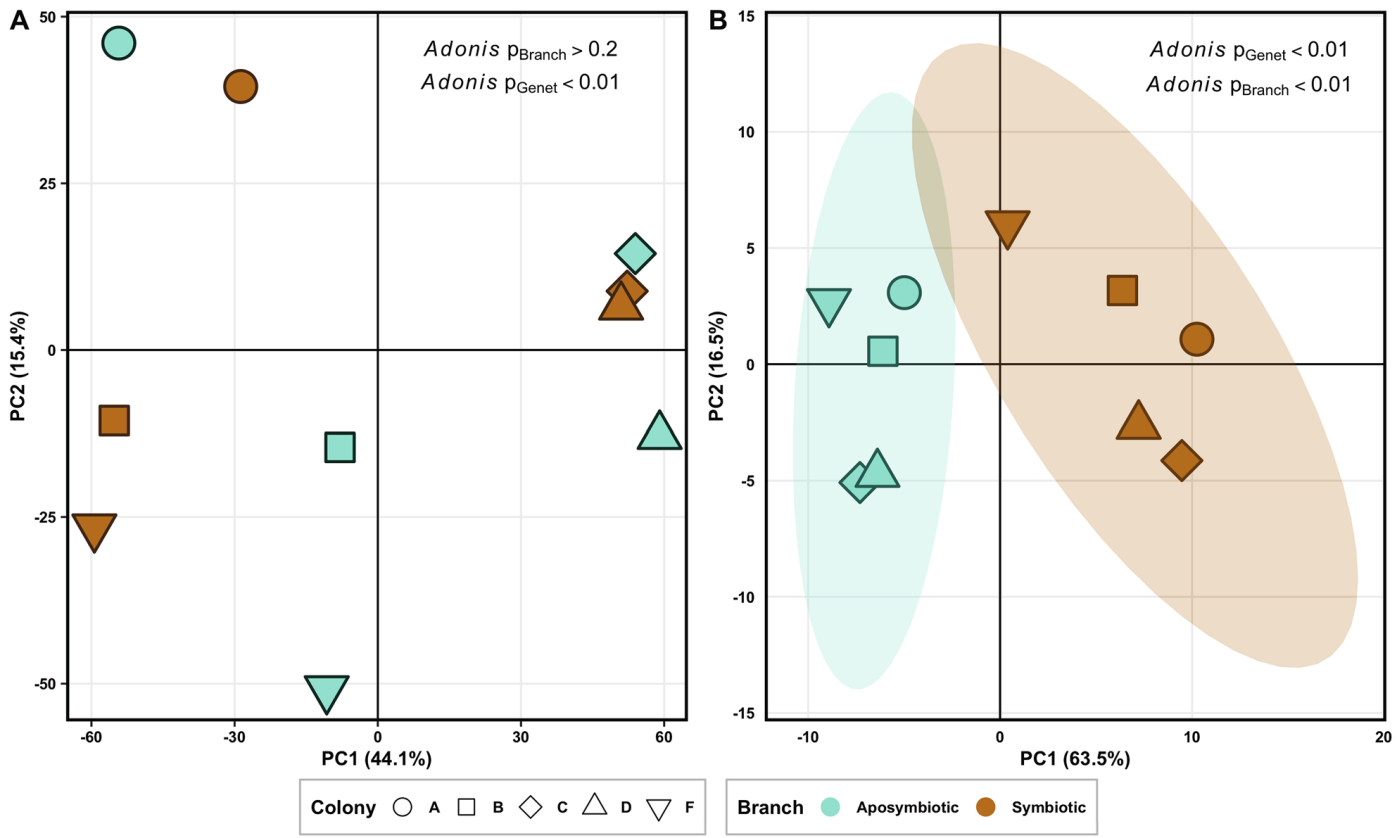
Principal component analyses of rlog transformed counts for (**A**) all genes and, (**B**) genes identified as differentially expressed by symbiotic state in *DESeq2* (N = 196, FDR < 0.1). PERMANOVA results for the effect of genetic background and symbiotic state (branch) are included.

Similar to other coral gene expression studies (e.g.^64,67^), most variation in gene expression was due to genet effects, which were found to be highly significant (*Adonis* PERMANOVA p value < 0.01) (Fig. 2). Influence of symbiotic state on overall gene expression was not significant (*p* = 0.23; Fig. 2A). Nevertheless, DESeq2 identified a small subset of 196 genes (< 1% of genes), that were differentially expressed (DEGs) in response to symbiotic state after accounting for genet effects (FDR = 0.1; Figure S3). When considering only DEGs, a clear separation between symbiotic and aposymbiotic samples is visible in principal component space (Fig. 2B). The majority of these DEGs (65%) were annotated, enabling the comparison of candidates with results from other studies and the use of gene ontology enrichment analyses to further explore the potential functional implications of symbiosis.

### Symbiotic branches showed significant upregulation of genes involved in nitrogen assimilation, and nutrient transport

**Figure 3:**
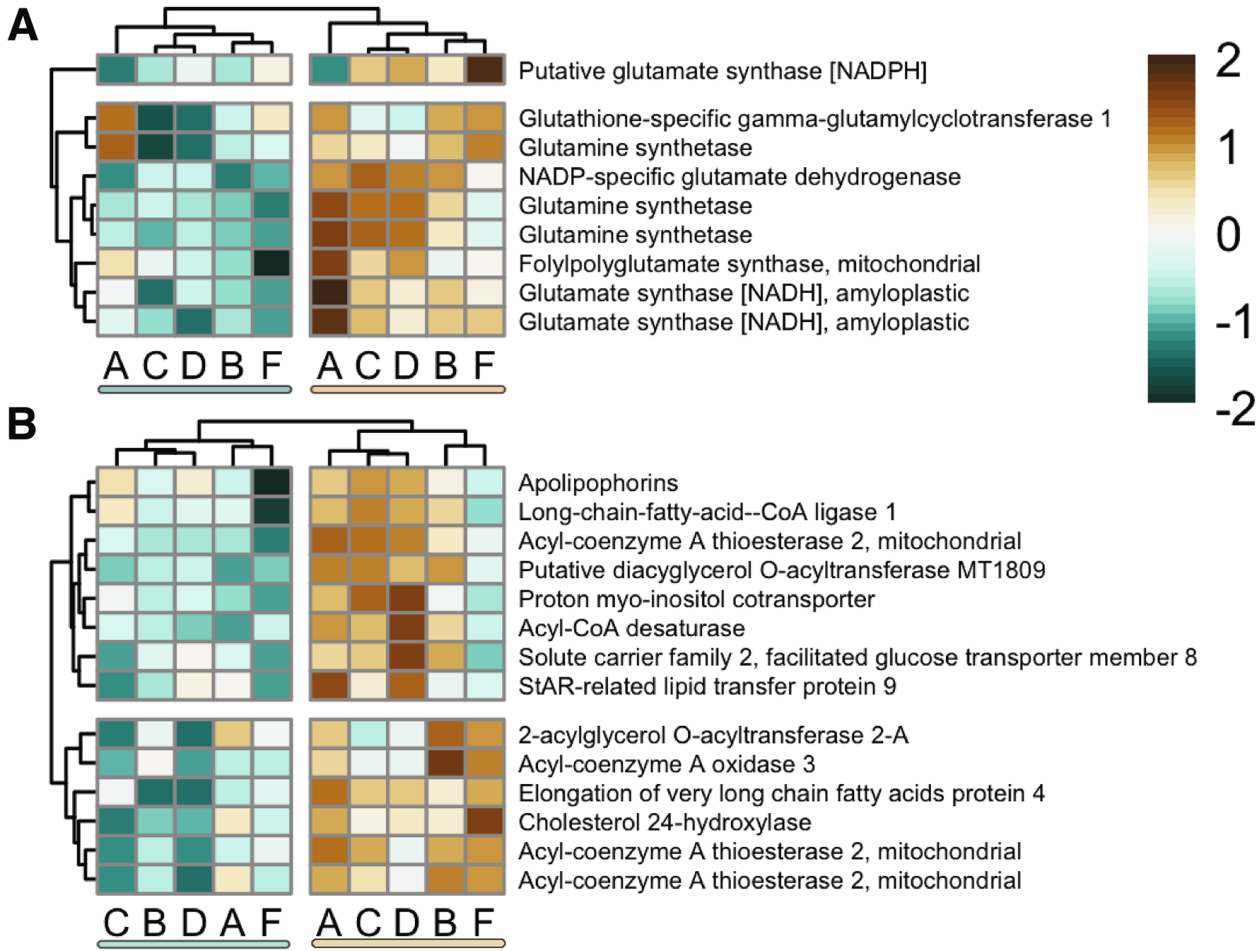
Significantly differentially expressed genes (FDR < 0.1) involved in glutamine production (**A**) and sugar or lipid transport/production (**B**). Color scale represents the gene’s log2 fold change. Colors are scaled within rows. Warm tones represent up-regulation (higher expression in symbiotic samples/lower in aposymbiotic) and cool tones down-regulation (lower expression in symbiotic samples/higher in aposymbiotic samples). Rows and columns are clustered hierarchically using Pearson correlation of their expression across genes and samples, respectively. Columns clearly clustered aposymbiotic and symbiotic samples. Genet ID is shown at the bottom of each column. Genes detailed here are also found in Figure S3, which shows a heatmap of all significantly differentially expressed genes.

Consistent with previous work (e.g.^68,69^), several genes involved in glutamate synthesis pathways, including glutamate and glutamine synthetases and glutamate dehydrogenases were all significantly upregulated in symbiotic branches (Fig. 3A). Glutamine synthetase (GS) and glutamine oxoglutarate aminotransferase (GOGAT) are recognized ammonium assimilation pathways across taxa, including plants^70,71^, bacteria and cyanobacteria^72^, insects^73^ and cnidarians^61,74^. For instance, glutamine synthetase, which uses glutamate and ammonium to produce glutamine, shows elevated expression in the coral *Pocillopora damicornis* after exposure to ammonium^75^. Glutamine synthetase, therefore, is thought to play a crucial role in ammonium/nitrogen recycling in intracellular symbioses across numerous taxa including cnidarians^61,69,75,76^, sponges^77^, insects^73,78^, clams^79^, and even salamanders^62^. In cnidarians, glutamine-dependent nitrogen cycling has been further implicated in host control of symbiont cell densities as a mechanism for controlling liable nitrogen available to the symbiont^61,69^. Further, glutamine was found to be a component of the coral “host factor”—a suite of compounds released by corals that were previously found to promote photosynthate transfer from symbiont cells to the host^80^. Elevated expression of glutamate/glutamine-associated genes in symbiotic branches corroborates past findings and suggests that with increasing symbiont densities, hosts are more closely regulating nitrogen cycling.

Several other genes associated with sugar and lipid transport or metabolism were also significantly upregulated in symbiotic branches, as would be expected given the nutritional exchanges that occur between coral hosts and their algal symbionts (Fig. 3B). For instance, the proteins apolipophorin, solute carrier family 2 facilitated glucose transport member 8 (SLC2A8), and proton myo-inositol cotransporter (SLC3A13) are responsible for the transport of various sugars^81^. All three of these genes show upregulation in symbiotic branches (Fig. 3A). SLC’s in particular, have been found to be differentially regulated under bleaching or during symbiosis establishment in corals^23,82^ and anemones^30,83^. Past studies on coral and anemone bleaching responses under elevated temperatures have found differential regulation of genes in acyl-coA pathways responsible for the formation and transport of fatty-acid changes (e.g.^82,84^). Here, we observed upregulation of genes associated with acyl-coA in symbiotic samples (Fig. 3B). In addition, we see that other lipid-related genes such as diacylglycerol O-acyltransferase (DGAT), cholesterol hydroxylase, lipid transfer protein, and fatty acid elongators are more highly expressed in symbiotic samples (Fig. 3B). Given that our samples were not subjected to stress-induced bleaching, these genes are likely key players in the nutritional exchanges between symbiotic partners and may be responsible for maintenance of symbiosis under baseline conditions.

### Enrichment of cell cycle regulation in symbiotic branches

Research on symbiont replication within the cnidarian host suggests that symbiont division is limited in symbiosis, as symbionts reared in culture media have higher replication rates than those *in hospite* (^35,66^; reviewed in^34^). Inhibition of the symbiont cell cycle and growth, through interference in the cell cycle G2/M checkpoint (during which the cell checks for DNA damage before proceeding to metaphase), is thought to be one of the ways in which the host can control symbiont cell density (reviewed in^34^). We find several cell cycle regulation gene ontology (GO) biological process terms, including “regulation of cell cycle G2/M phase transition”, “negative regulation of cell phase transition”, “G1/S transition”, and “cell cycle arrest” to be significantly enriched among genes that were upregulated in symbiotic branches (Figure S4). Molecular function terms for “damaged DNA binding”, “chromatin binding”, and cellular components terms for “DNA repair complex”, “site of DNA damage”, “kinetochore”, along with many similar terms in both categories were also found to be upregulated in symbiotic branches (Figure S5–S6). In addition to observed GO enrichment, several differentially expressed genes, including Hemicentin-1, which has been found to be important for mitotic spindle formation^85^, DNA lyase (which repairs DNA), and DNA helicases were significantly upregulated in symbiotic branches (Figure S3). This implies symbiotic branches are leveraging cell cycle control as a primary mechanism to regulate symbiont population density.

**Figure 4:**
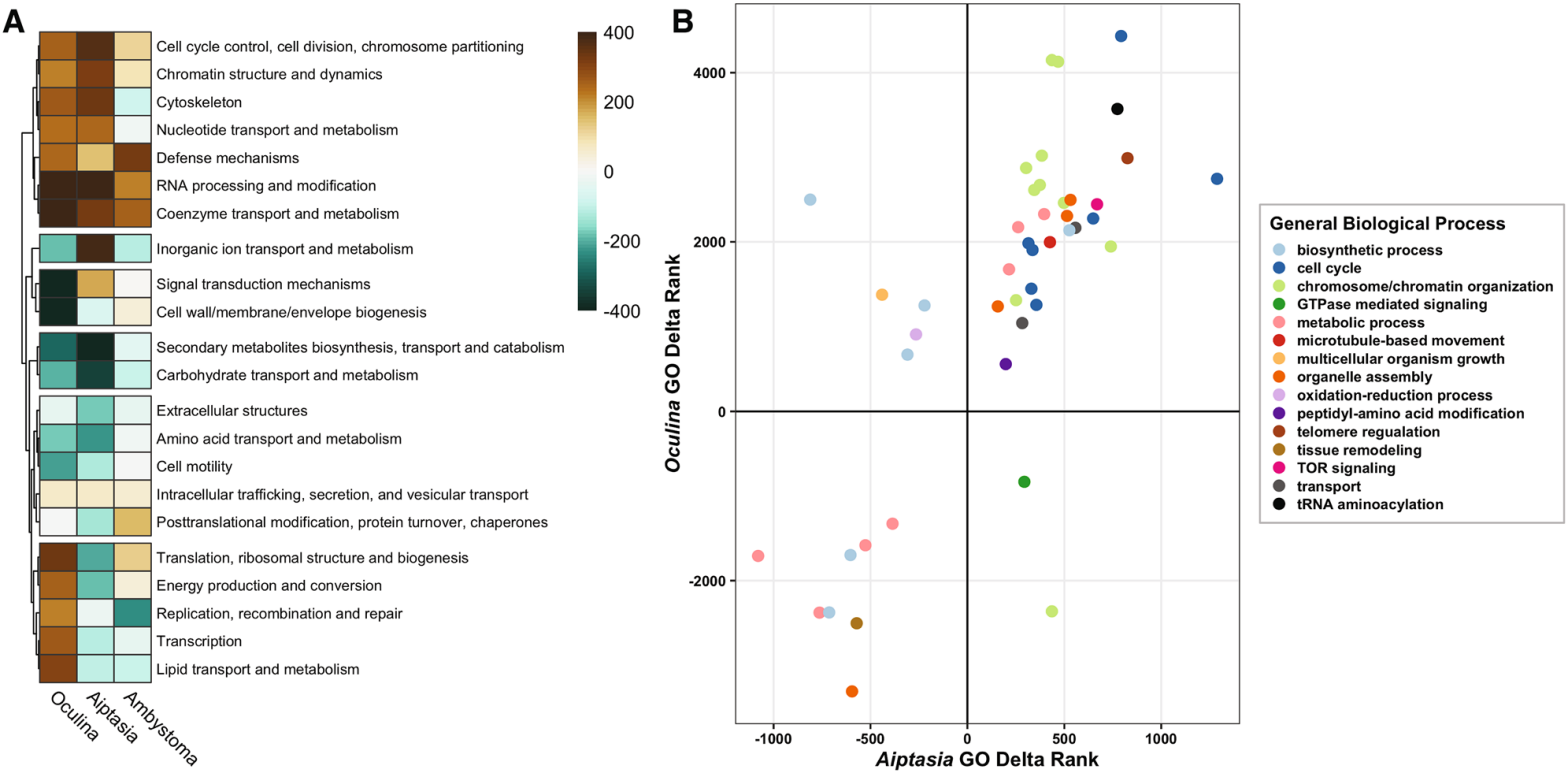
KOG (left) and GO delta rank (right) comparisons of gene expression differences between aposymbiotic and symbiotic branches in *Oculina arbuscula* (current study), *Exaiptasia pallida* (Aiptasia; Cui et al.^61^), and the salamander *Ambystoma maculatum* (Burns et al.^62^). (**A**) Delta rank values of KOG classes of gene expression for each dataset based on signed -ln(p values) for differential expression. Warm tones (positive rank values) correspond to classes that were upregulated (higher expression in symbiotic samples). Cool tones (negative rank values) correspond to classes that were downregulated (higher expression in aposymbiotic samples). (**B**) Delta rank values of GO biological process terms that were significantly enriched (p value < 0.05) in both *O. arbuscula* and *E. pallida* (Aiptasia) datasets. Positive ranks are GO terms that were associated with upregulated genes (higher expression in symbiotic samples), while negative ranks were downregulated. Colors of points correspond to a simplified biological process category for visualization. See Supplementary Table S5 for the full labels of terms represented in the figure.

Despite the deep phylogenetic divergence between cnidarians and amphibians, KOG comparisons between *O. arbuscula*, *E. pallida*, and *A. maculatum* were surprisingly consistent across most categories (Fig. 4A). “Cell cycle control” was upregulated across all three taxa (Fig. 4A). Comparison of biological process terms through GO delta rank comparisons between *O. arbuscula* and *E. pallida* also showed consistent patterns in many terms associated with cell cycle control (Fig. 4B), underscoring its role in maintenance of symbiosis across various taxa. A mechanistic target of rapamycin (mTOR) GO term was also found to be enriched among genes upregulated in both *O. arbuscula* and *E. pallida* (Fig. 4B). mTOR is a key player in pathways that control cell growth; differences in mTOR expression have also been observed in aphid bacteriocytes hosting higher titers of the aphid bacteria symbiont *Buchera *sp.^86^. Other terms including “RNA processing”, “coenzyme transport and metabolism”, “chromatin structure and dynamics” were also consistently upregulated in symbiotic samples across all three taxa (Fig. 4A). These patterns suggest that endosymbiosis may rely on a conserved set of responses by the host organism that enables control of the symbiont cell density through regulation of cell cycle checkpoints as well as necessary nutrient and metabolic exchanges between host and symbiont cells.

### Increased expression of exocytosis and immune response-related genes in aposymbiotic branches

Alternative methods for controlling symbiont densities are exocytosis/vomocytosis or induced apoptosis of symbiont-bearing cells^31,34^. Interestingly, aposymbiotic branches showed enrichment of several biological process terms associated with and including “regulation of exocytosis;” and cellular components terms for “secretory vesicles” that were upregulated in aposymbiotic branches (Table S4; Figure S6). In addition, “MAPK cascade-” associated biological process terms and “frizzled” molecular function terms were also found to be enriched in aposymbiotic branches (Tables S2, S3; Figures S4, S5). MAPK signaling can induce cell apoptosis (reviewed in^87^), as can binding to *frizzled* receptors^88^. In addition, cytochrome-c, which can mediate cell apoptosis (reviewed in^89^), is significantly upregulated in aposymbiotic samples (Figure S3). It appears that aposymbiotic coral polyps may, therefore, be expelling greater numbers of symbionts through apoptosis or active secretion as opposed to attempting to control densities through mechanisms such as nitrogen limitation or cell cycle disruption; though, this hypothesis remains to be tested.

Recent work on the uptake and release of different species of microalgae by *Aiptasia* larvae showed expulsion of unwanted microalgae occurs primarily through vomocytosis—a process that is likely controlled through host innate immune pathways^31^. Symbiodiniaceae may escape this vomocytosis through suppression of toll-like receptor and MyD88 pathways of the host’s immune system^31^. Other studies looking at the immune system regulator NF-KB (which interacts with MyD88) have found that symbiosis establishment can prompt widespread inhibition of expression of NF-KB in Aiptasia^38,90–92^ as well as other taxa, including corals^93^, salamanders^62^, and sponges^77^. We observe extensive enrichment of immune system biological process terms in aposymbiotic branches including, though not limited to, “NF-KB regulation”, “inflammatory response”, “complement activation”, “activation of innate immune response,” and “regulation of tumor necrosis factor superfamily cytokine production”, as well as molecular function terms for “cytokine receptor binding” and “tumor necrosis factor binding” (Table S3; Figures S4, S5). In addition, immune-associated genes, such as toll-like receptor 2, tenascin (which can interact with TLRs to prompt inflammatory responses^94,95^ are significantly upregulated in aposymbiotic branches (Figure S3). Our findings here align with other work and suggest that aposymbiotic branches have higher immune response activity than symbiotic branches.

Whether the reduction in symbiont densities re-activates the coral immune system, or if activation of the coral immune system prompts greater symbiont expulsion through vomocytotic/apoptotic mechanisms is not yet entirely clear. Prior work^31^, stimulated Aiptasia larvae with LPS to prompt an immune response, which consequently increased the number of expelled symbionts, suggesting that immune system activation prompts symbiont expulsion. This relationship, however, depended on the timing of LPS exposure relative to symbiont infection; only concurrent LSP exposure and symbiont infection led to later symbiont expulsion^31^. Thus, it is still unclear what immune system feedback(s) may prompt the disruption of already established symbiosis. Disentangling these causal links, however, would be incredibly valuable for coral reef science given that corals globally are experiencing increasing mortality from diseases^96,97^. A recent epidemic outbreak of a novel disease called stony coral tissue loss disease (SCTLD) has been rampantly spreading through the Caribbean and causing devastating losses^98–101^. If symbiosis with specific Symbiodiniaceae species incur different immunological trade-offs as some research suggests (e.g.^31,38,93^), this may have vast implications for Symbiodiniaceae-host associations in the context of increasing oceanic temperatures (where more thermally tolerant Symbiodiniaceae strains may be preferable) and coral disease susceptibility and response.

Interestingly, in the KOG comparisons across taxa, “defense mechanisms” showed upregulation in symbiotic samples. This contrasts with our GO ontology results for *O. arbuscula* and is surprising given the previous studies discussed above. Upon closer inspection, however, only a handful of genes were annotated with this KOG category (21–32 genes, depending on the taxa), and most of these were not canonical immune response genes (for instance, no toll-like receptors, NF-KB-associated genes, immunoglobulins, tumor necrosis factors, or melanin pathway genes were among the 31 genes annotated with “defense mechanisms” in *O. arbuscula*). This underscores a potential weakness of KOG classes and KOG enrichment analysis: genes are only assigned one KOG class and due to the inherent broadness of the classes, a given designation may not be the most representative of gene function in the organism of interest.

### *Oculina arbuscula* can serve as a robust model system for understanding endosymbiosis

Despite some limitations, the remarkable agreement of KOG enrichment across divergent taxa suggests conserved gene regulation in the presence of endosymbionts across the animal kingdom. Future studies that more closely examine molecular and physiological changes across symbiotic/aposymbiotic samples or tissues may elucidate key genes and pathways responsible for the sustained mutualistic associations between a variety of photosynthetic microalgae and their metazoan hosts. In particular, studying such pathways in the absence of additional stressors (such a high temperatures to induce bleaching) through the use of facultative models like *O. arbuscula* or *E. pallida* or through newer techniques like spatial transcriptomics or single-cell RNA-seq will undoubtedly uncover differences between baseline maintenance of symbiosis and stress-induced dysbiosis, allowing us to better understand how at-risk organisms—such as reef-building corals—regulate symbiosis in the face of multiple stressors.

In this work, we compare gene expression of aposymbiotic and symbiotic branches of the same colonies of the facultative coral *O. arbuscula* and find support for the regulation of symbiotic density through cell cycle regulation and nitrogen cycling in symbiotic branches. Meanwhile, aposymbiotic branches appear to achieve low symbiont density through active secretion mechanisms such as exocytosis or through apoptosis of symbiont-containing cells. We also find that aposymbiotic branches have upregulation of immune-related pathways, which is consistent with previous work. Whether higher immune response is the cause of increased symbiont expulsion, or a consequence of lower symbiont density will require further study.

We also showcase that the calcifying coral *O. arbuscula* can serve as a useful model for studying endosymbiosis (Fig. 1B). Its large colony size allows for numerous genotypic replicates through colony fragmentation, while its ability to host symbionts in a facultative manner, has allowed us to pinpoint several key features of gene expression that are important for symbiosis maintenance. These responses can then be teased apart from the much larger repertoire of genes that are differentially expressed during stress-induced dysbiosis such as temperature-induced bleaching. An additional feature of *O. arbuscula* is that colonies can be maintained in a fully aposymbiotic state, after induction of bleaching with the use of menthol (Rivera and Davies, unpublished data), allowing future work to compare the energetic and physiological differences between symbiotic and aposymbiotic fragments while explicitly controlling for genetic background. Such comparisons have the potential to reveal direct genetic underpinnings and cellular mechanisms of symbiosis, which are challenging to disentangle in systems that exhibit natural variation in symbiotic densities. Future work on this and other facultative coral systems such as *Astrangia poculata*^44,102^ or *Oculina patagonica*^103,104^ will help elucidate the workings of a canonical and ecologically important endosymbiosis between reef-building corals and their dinoflagellate partners.

## Supplementary Information

Supplementary Information 1.

Supplementary Information 2.

Supplementary Information 3.

Supplementary Information 4.

Supplementary Information 5.

Supplementary Information 6.

Supplementary Information 7.

Supplementary Information 8.

Supplementary Information 9.

## Data Availability

Raw reads for all 12 samples (6 colonies, aposymbiotic and symbiotic branches) are available on the NCBI Short Read Archive (SRA) under BioProject number PRJNA746598. All other scripts and code required to generate the transcriptome assembly are hosted at https://github.com/hrivera28/Oculina_arbuscula_transcriptome. Scripts and input data for all other analyses, e.g. read mapping, differential expression, etc. are hosted at https://github.com/hrivera28/oculina_apo-sym_gene_expression. Metadata for samples and all other information and figures referenced in the text is supplied in the supplementary materials or main manuscript.
