# Supplementary material for: Symbiosis maintenance in the facultative coral, Oculina arbuscula, relies on nitrogen cycling, cell cycle modulation, and immunity
Source: Sci Rep. 2021 Oct 27;11:21226. doi: 10.1038/s41598-021-00697-6 (PMC8551165; doi:10.1038/s41598-021-00697-6)
Supplement: Supplementary file 6 — Supplementary Information 6. [file 41598_2021_697_MOESM6_ESM.docx]

Supplementary Figures


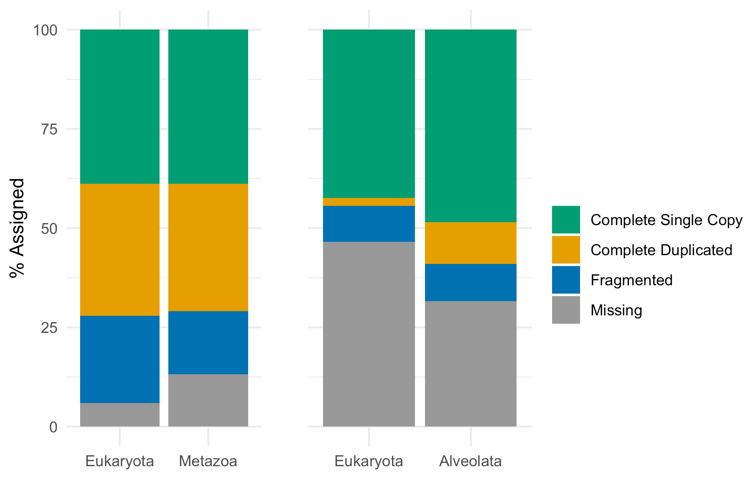


**Figure S1.** Percentage of core BUSCO genes in various gene sets that were found in assembled *O. arbuscula* and *B. psygmophilum* transcriptomes. Both transcriptomes were matched to the eukaryota gene set. In addition, *O. arbuscula* was also matched to the metazoa gene set, and *B. psygmophium* to the alveolata gene set. The coral transcriptome contains a much higher proportion of the genes in BUSCO core eukaryota sets (>90%), while the symbiont transcriptome is more incomplete (46% missing), though results were better (only 31% missing) when considering the alveolata gene set (dinoflagellates are alveolates).


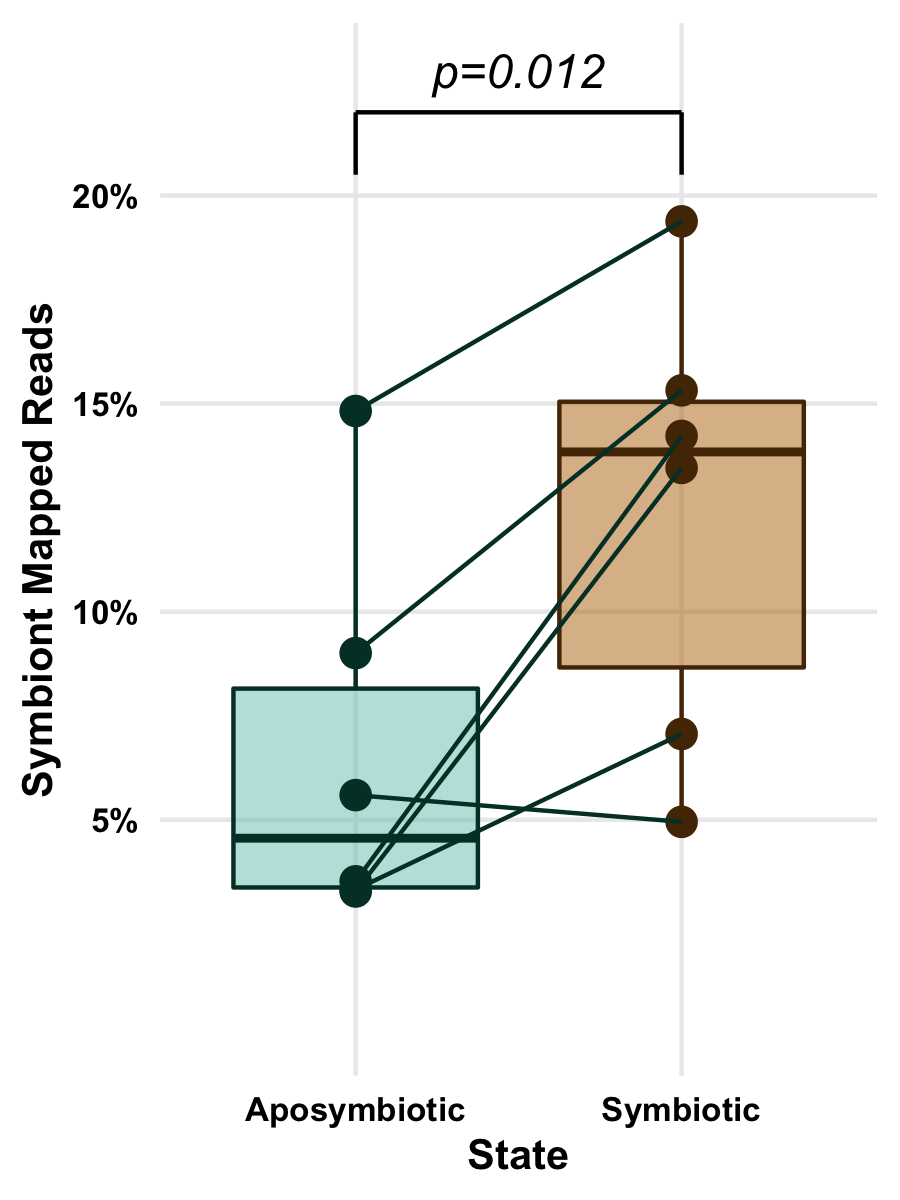


**Figure S2.** Percent of reads mapping to *B. psygmophilum* (symbiont) transcriptome for symbiotic and aposymbiotic coral branches from the same colony (points connected by lines). Aposymbiotic samples had significantly lower mapping efficiencies to the symbiont transcriptome when compared to reads mapping to the *Oculina arbuscula* transcriptome (*p*=0.012; one-tailed paired t-test, *N*=6).

**
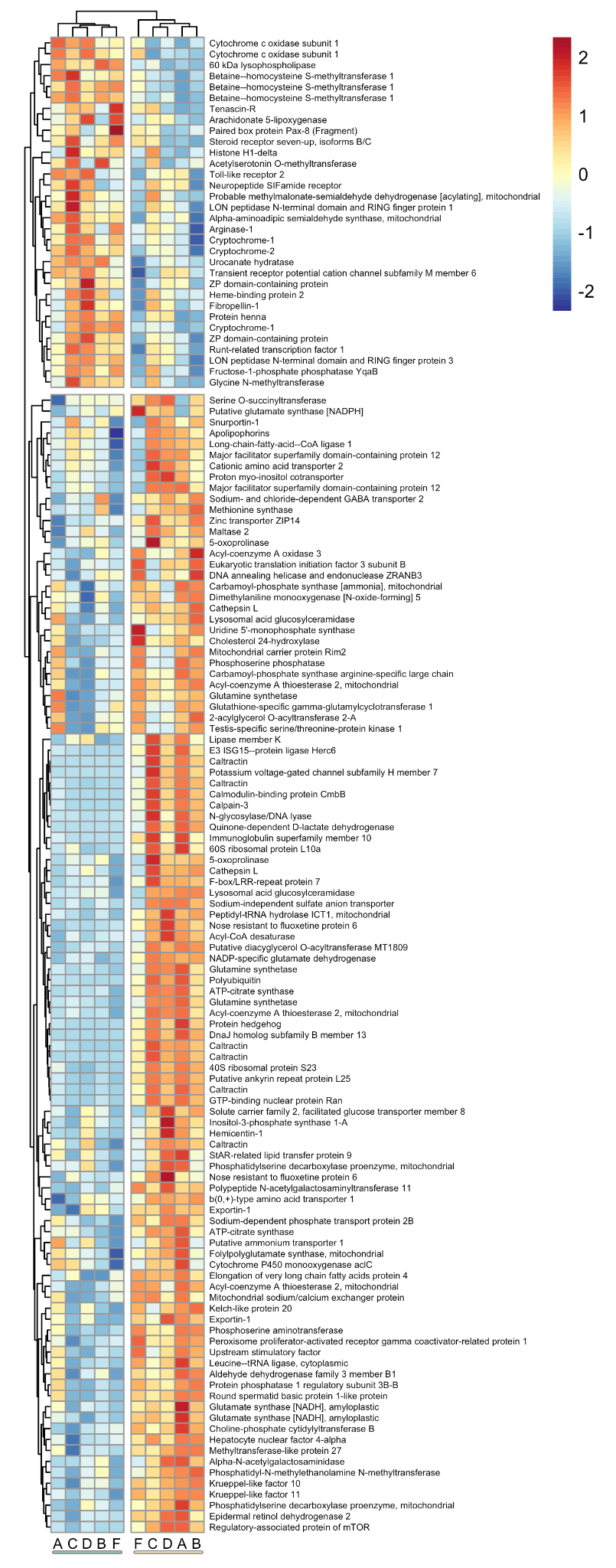
**

**Figure S3.** Heatmap of significantly differentially expressed genes identified by *DESeq2* (FDR<0.1). Unannotated genes are omitted for visualization purposes. Rows are genes and columns are samples. Color scale is the log2 fold change. Colors are scaled within rows. Warm tones represent up-regulation (higher expression in symbiotic samples/lower in aposymbiotic) and cool tones down-regulation (lower expression in symbiotic samples/higher in aposymbiotic samples). Rows and columns are clustered hierarchically using Pearson correlation of their expression across genes and samples, respectively. Columns clustered with all aposymbiotic samples (teal bar) on the left and all symbiotic (brown bar) samples on the right. Genet ID is shown at the bottom of each column.

*Submitted as a separate file due to size*

**Figure S4.** GO enrichment of biological process terms based on signed -log p-values of gene expression data between aposymbiotic and symbiotic *Oculina arbuscula* branches. Brown colors represent terms that showed overall enrichment among genes that are upregulated in symbiotic branches, teal colors represent terms enriched among genes that were up-regulated in aposymbiotic branches. The size/type of the font corresponds to the p-value associated with the enrichment of those terms based on Mann-Whitney U-test. The numbers in front of each term correspond to the number of genes annotated with that GO term that had an unadjusted p-value of 0.05 for differential expression between aposymbiotic/symbiotic branches over all the genes in the dataset that were annotated with that GO term. For a full explanation of GOMWU see (<https://github.com/z0on/GO_MWU>).

*Submitted as a separate file due to size*

**Figure S5.** GO enrichment of molecular function terms based on signed -log p-values of gene expression data between aposymbiotic and symbiotic *Oculina arbuscula* branches. Brown colors represent terms that showed overall enrichment among genes that are upregulated in symbiotic branches, teal colors represent terms enriched among genes that were up-regulated in aposymbiotic branches. The size/type of the font corresponds to the p-value associated with the enrichment of those terms based on Mann-Whitney U-test. The numbers in front of each term correspond to the number of genes annotated with that GO term that had an unadjusted p-value of 0.05 for differential expression between aposymbiotic/symbiotic branches over all the genes in the dataset that were annotated with that GO term. For a full explanation of GOMWU see (<https://github.com/z0on/GO_MWU>).

*Submitted as a separate file due to size*

**Figure S6.** GO enrichment of cellular component terms based on signed -log p-values of gene expression data between aposymbiotic and symbiotic *Oculina arbuscula* branches. Brown colors represent terms that showed overall enrichment among genes that are upregulated in symbiotic branches, teal colors represent terms enriched among genes that were up-regulated in aposymbiotic branches. The size/type of the font corresponds to the p-value associated with the enrichment of those terms based on Mann-Whitney U-test. The numbers in front of each term correspond to the number of genes annotated with that GO term that had an unadjusted p-value of 0.05 for differential expression between aposymbiotic/symbiotic branches over all the genes in the dataset that were annotated with that GO term. For a full explanation of GOMWU see (<https://github.com/z0on/GO_MWU>).

**
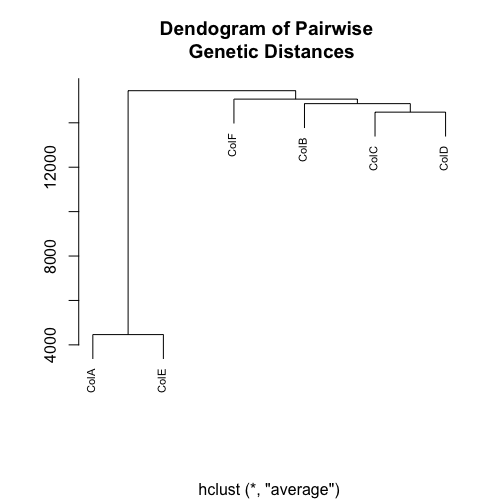
**

**Figure S7.** Dendrogram (hierarchical clustering) of SNP data from RNA-seq reads. Colony A and E show much higher genetic relatedness, strongly suggesting that they are potential clonemates.

**
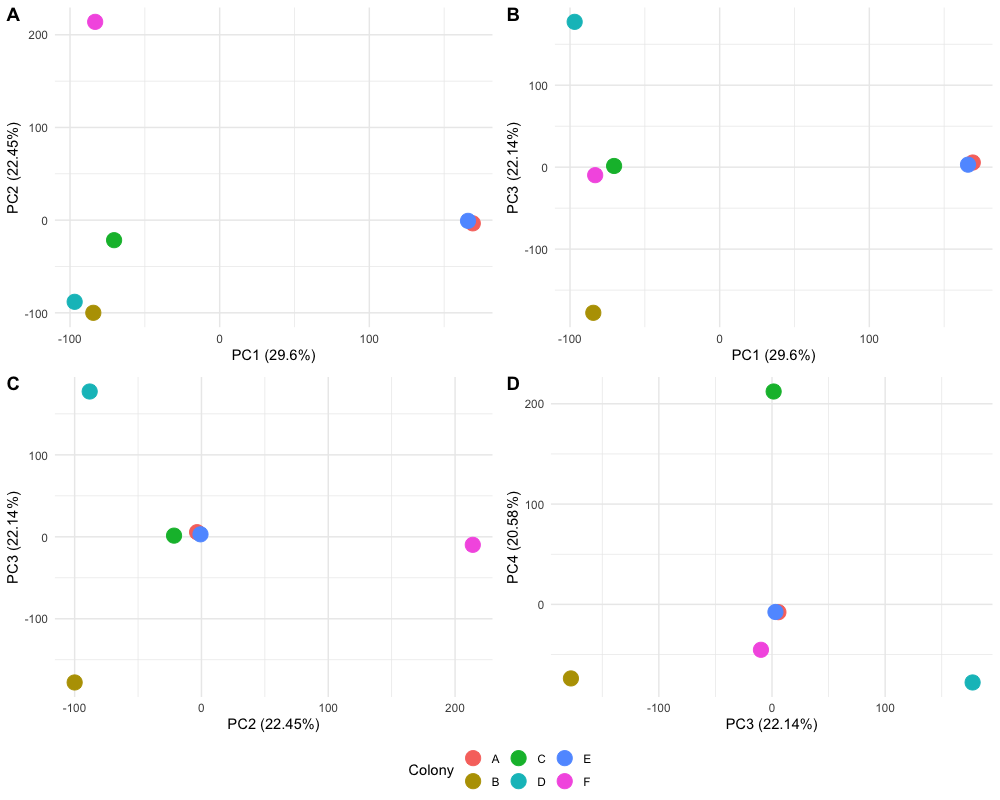
**

**Figure S8.** Principal component analyses of SNP data from RNA-seq reads. Across several principal components, that cumulatively explain >93% of the variation colonies A and E always overlap in PC space, indicating that they have nearly identical genetic composition and are potential clonemates.

Supplementary Table Legends

**Table S1.** Excel spreadsheet enumerating number of raw reads and quality scores for each sample (‘RawReads’ tab) and the percentage/number of reads mapping to the coral host and symbiont transcriptomes for each sample (‘MappingStats’ tab).

**Table S2.** List of biological process terms displayed in Figure S4, where 1 corresponds to up-regulation in symbiotic branches and 0 corresponds to up-regulation in aposymbiotic branches (equivalently down-regulation in symbiotic branches). Terms with a direction of 1 appear in brown/warm toned text in Figure S4, and those with a direction of 0 appear in teal/cool toned text. Provided to enable searchability of GO terms for readers.

**Table S3.** List of molecular function terms displayed in Figure S5, where 1 corresponds to up-regulation in symbiotic branches and 0 corresponds to up-regulation in aposymbiotic branches (equivalently down-regulation in symbiotic branches). Terms with a direction of 1 appear in brown/warm toned text in Figure S4, and those with a direction of 0 appear in teal/cool toned text. Provided to enable searchability of GO terms for readers.

**Table S4.** List of cellular component terms displayed in Figure S6, where 1 corresponds to up-regulation in symbiotic branches and 0 corresponds to up-regulation in aposymbiotic branches (equivalently down-regulation in symbiotic branches). Terms with a direction of 1 appear in brown/warm toned text in Figure S4, and those with a direction of 0 appear in teal/cool toned text. Provided to enable searchability of GO terms for readers.

**Table S5.** Key for shortened ‘General biological process’ terms displayed in Figure 4B.
