# Supplementary figures and images for: Symbiosis maintenance in the facultative coral, Oculina arbuscula, relies on nitrogen cycling, cell cycle modulation, and immunity
Source: Sci Rep. 2021 Oct 27;11:21226. doi: 10.1038/s41598-021-00697-6 (PMC8551165; doi:10.1038/s41598-021-00697-6)

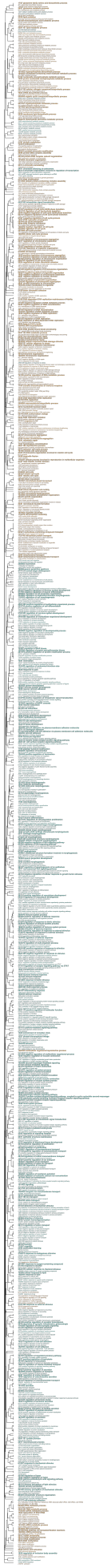

Supplement: Supplementary file 7 — Supplementary Information 7. [file 41598_2021_697_MOESM7_ESM.pdf]

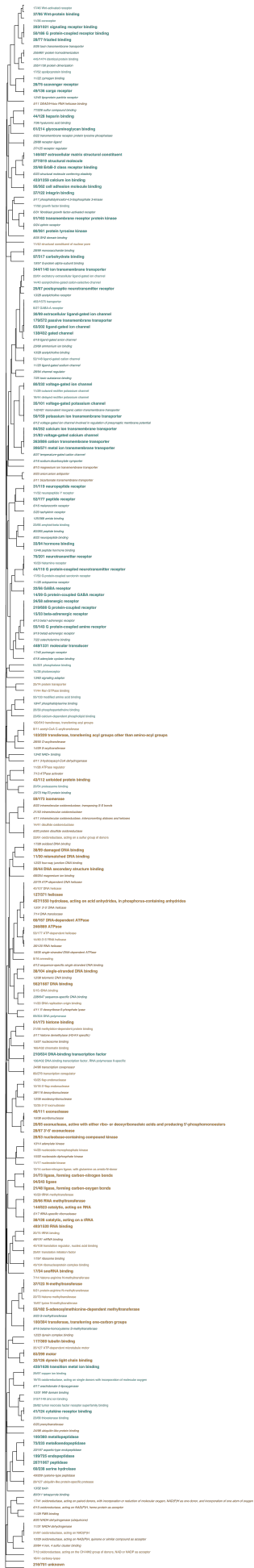

2 x 10<sup>4</sup>  
2 x 10<sup>3</sup>  
2 x 10<sup>2</sup>

Supplement: Supplementary file 8 — Supplementary Information 8. [file 41598_2021_697_MOESM8_ESM.pdf]
