# Supplementary material for: Symbiosis maintenance in the facultative coral, Oculina arbuscula, relies on nitrogen cycling, cell cycle modulation, and immunity
Source: Sci Rep. 2021 Oct 27;11:21226. doi: 10.1038/s41598-021-00697-6 (PMC8551165; doi:10.1038/s41598-021-00697-6)
Supplement: Supplementary file 9 — Supplementary Information 9. [file 41598_2021_697_MOESM9_ESM.pdf]

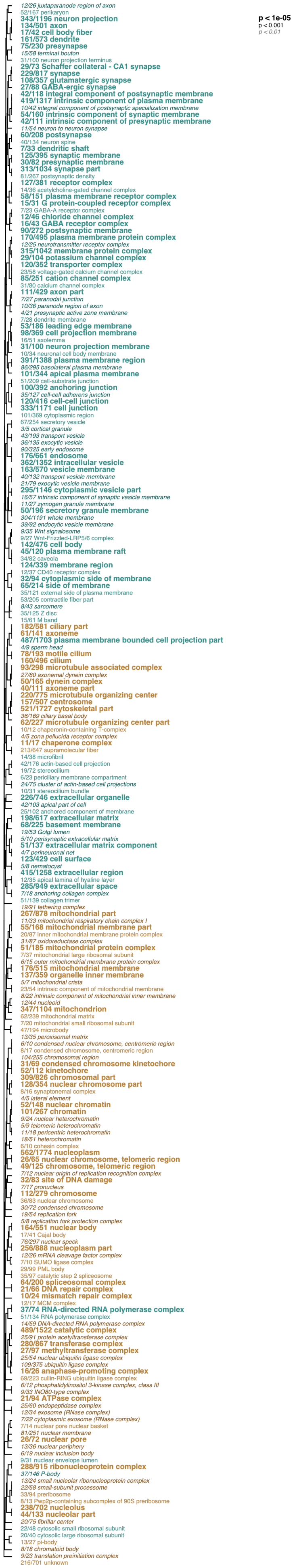

p < 1e-05
p < 0.001
p < 0.01
12/26 juxtaparanode region of axon
52/167 perikaryon
343/1196 neuron projection
134/501 axon
17/42 cell body fiber
161/573 dendrite
75/230 presynapse
15/58 terminal bouton
31/100 neuron projection terminus
29/73 Schaffer collateral - CA1 synapse
229/817 synapse
108/357 glutamatergic synapse
27/88 GABA-ergic synapse
42/118 integral component of postsynaptic membrane
419/1317 intrinsic component of plasma membrane
10/42 integral component of postsynaptic specialization membrane
54/160 intrinsic component of synaptic membrane
42/111 intrinsic component of presynaptic membrane
11/54 neuron to neuron synapse
60/208 postsynapse
40/134 neuron spine
7/33 dendritic shaft
125/395 synaptic membrane
30/82 presynaptic membrane
313/1034 synapse part
81/267 postsynaptic density
127/381 receptor complex
14/36 acetylcholine-gated channel complex
58/151 plasma membrane receptor complex
15/31 G protein-coupled receptor complex
7/23 GABA-A receptor complex
12/46 chloride channel complex
16/43 GABA receptor complex
90/272 postsynaptic membrane
170/495 plasma membrane protein complex
12/25 neurotransmitter receptor complex
315/1042 membrane protein complex
29/104 potassium channel complex
120/352 transporter complex
23/58 voltage-gated calcium channel complex
85/251 cation channel complex
31/80 calcium channel complex
111/429 axon part
7/27 paranodal junction
10/36 paranode region of axon
4/21 presynaptic active zone membrane
7/28 dendrite membrane
53/186 leading edge membrane
98/369 cell projection membrane
16/51 axolemma
31/100 neuron projection membrane
10/34 neuronal cell body membrane
391/1388 plasma membrane region
86/295 basolateral plasma membrane
101/344 apical plasma membrane
51/209 cell-substrate junction
100/392 anchoring junction
35/127 cell-cell adherens junction
120/416 cell-cell junction
333/1171 cell junction
101/369 cytoplasmic region
67/254 secretory vesicle
3/5 cortical granule
43/193 transport vesicle
36/135 exocytic vesicle
90/325 early endosome
176/661 endosome
362/1352 intracellular vesicle
163/570 vesicle membrane
40/132 transport vesicle membrane
21/79 exocytic vesicle membrane
295/1146 cytoplasmic vesicle part
16/57 intrinsic component of synaptic vesicle membrane
11/27 zymogen granule membrane
50/196 secretory granule membrane
304/1191 whole membrane
39/92 endocytic vesicle membrane
9/35 Wnt signalosome
9/27 Wnt-Frizzled-LRP5/6 complex
142/476 cell body
45/120 plasma membrane raft
34/82 caveola
124/339 membrane region
12/37 CD40 receptor complex
32/94 cytoplasmic side of membrane
65/214 side of membrane
35/121 external side of plasma membrane
53/205 contractile fiber part
8/43 sarcomere
35/125 Z disc
15/61 M band
182/581 ciliary part
61/141 axoneme
487/1703 plasma membrane bounded cell projection part
4/9 sperm head
78/193 motile cilium
160/496 cilium
93/298 microtubule associated complex
27/80 axonemal dynein complex
50/165 dynein complex
40/111 axoneme part
220/775 microtubule organizing center
157/507 centrosome
521/1727 cytoskeletal part
36/169 ciliary basal body
62/227 microtubule organizing center part
10/12 chaperonin-containing T-complex
4/5 zona pellucida receptor complex
11/17 chaperone complex
213/647 supramolecular fiber
14/38 microfibril
42/176 actin-based cell projection
19/72 stereocilium
6/23 periciliary membrane compartment
24/75 cluster of actin-based cell projections
10/31 stereocilium bundle
226/746 extracellular organelle
42/103 apical part of cell
25/102 anchored component of membrane
198/617 extracellular matrix
68/225 basement membrane
19/53 Golgi lumen
5/10 perisynaptic extracellular matrix
51/137 extracellular matrix component
4/7 perineuronal net
123/429 cell surface
5/8 nematocyst
415/1258 extracellular region
12/35 apical lamina of hyaline layer
285/949 extracellular space
7/18 anchoring collagen complex
51/139 collagen trimer
19/91 tethering complex
267/878 mitochondrial part
11/33 mitochondrial respiratory chain complex I
55/168 mitochondrial membrane part
20/87 inner mitochondrial membrane protein complex
31/87 oxidoreductase complex
51/185 mitochondrial protein complex
7/37 mitochondrial large ribosomal subunit
6/15 outer mitochondrial membrane protein complex
176/515 mitochondrial membrane
137/359 organelle inner membrane
5/7 mitochondrial crista
23/54 intrinsic component of mitochondrial membrane
8/22 intrinsic component of mitochondrial inner membrane
12/44 nucleoid
347/1104 mitochondrion
62/239 mitochondrial matrix
7/20 mitochondrial small ribosomal subunit
47/194 microbody
13/35 peroxisomal matrix
6/10 condensed nuclear chromosome, centromeric region
8/17 condensed chromosome, centromeric region
104/255 chromosomal region
31/69 condensed chromosome kinetochore
52/112 kinetochore
309/826 chromosomal part
128/354 nuclear chromosome part
8/16 synaptonemal complex
4/5 lateral element
52/148 nuclear chromatin
101/267 chromatin
9/24 nuclear heterochromatin
5/9 telomeric heterochromatin
11/18 pericentric heterochromatin
18/51 heterochromatin
6/10 cohesin complex
562/1774 nucleoplasm
26/65 nuclear chromosome, telomeric region
49/125 chromosome, telomeric region
7/12 nuclear origin of replication recognition complex
32/83 site of DNA damage
7/17 pronucleus
112/279 chromosome
36/83 nuclear chromosome
30/72 condensed chromosome
19/54 replication fork
5/8 replication fork protection complex
164/551 nuclear body
17/41 Cajal body
76/297 nuclear speck
256/888 nucleoplasm part
12/26 mRNA cleavage factor complex
7/10 SUMO ligase complex
29/99 PML body
35/97 catalytic step 2 spliceosome
64/200 spliceosomal complex
21/66 DNA repair complex
10/24 mismatch repair complex
12/17 MCM complex
37/74 RNA-directed RNA polymerase complex
51/134 RNA polymerase complex
14/59 DNA-directed RNA polymerase complex
489/1522 catalytic complex
25/91 protein acetyltransferase complex
280/867 transferase complex
27/97 methyltransferase complex
25/54 nuclear ubiquitin ligase complex
109/375 ubiquitin ligase complex
16/26 anaphase-promoting complex
69/223 cullin-RING ubiquitin ligase complex
6/12 phosphatidylinositol 3-kinase complex, class III
9/33 INO80-type complex
21/94 ATPase complex
25/60 endopeptidase complex
12/34 exosome (RNase complex)
7/22 cytoplasmic exosome (RNase complex)
7/14 nuclear pore nuclear basket
81/251 nuclear membrane
26/72 nuclear pore
13/36 nuclear periphery
6/19 nuclear inclusion body
9/31 nuclear envelope lumen
288/915 ribonucleoprotein complex
37/146 P-body
13/24 small nucleolar ribonucleoprotein complex
22/58 small-subunit processome
33/94 preribosome
8/13 Pwp2p-containing subcomplex of 90S preribosome
238/702 nucleolus
44/133 nucleolar part
20/75 fibrillar center
22/48 cytosolic small ribosomal subunit
20/40 cytosolic large ribosomal subunit
13/27 pi-body
8/18 chromatoid body
9/23 translation preinitiation complex
216/701 unknown
